# Ketamine: differential neurophysiological dynamics in functional networks in the rat brain

**DOI:** 10.1038/tp.2017.198

**Published:** 2017-09-19

**Authors:** A Ahnaou, H Huysmans, R Biermans, N V Manyakov, W H I M Drinkenburg

**Affiliations:** 1 Department of Neuroscience Discovery, Janssen Research & Development, A Division of Janssen Pharmaceutica NV, Beerse, Belgium

## Abstract

Recently, the *N*-methyl-d-aspartate-receptor (NMDAR) antagonist ketamine has emerged as a fast-onset mechanism to achieve antidepressant activity, whereas its psychomimetic, dissociative and amnestic effects have been well documented to pharmacologically model schizophrenia features in rodents. Sleep–wake architecture, neuronal oscillations and network connectivity are key mechanisms supporting brain plasticity and cognition, which are disrupted in mood disorders such as depression and schizophrenia. In rats, we investigated the dynamic effects of acute and chronic subcutaneous administration of ketamine (2.5, 5 and 10 mg kg^−1^) on sleep–wake cycle, multichannels network interactions assessed by coherence and phase–amplitude cross-frequency coupling, locomotor activity (LMA), cognitive information processing as reflected by the mismatch negativity-like (MMN) component of event-related brain potentials (ERPs). Acute ketamine elicited a short, lasting inhibition of rapid eye movement (REM) sleep, increased coherence in higher gamma frequency oscillations independent of LMA, altered theta-gamma phase–amplitude coupling, increased MMN peak-amplitude response and evoked higher gamma oscillations. In contrast, chronic ketamine reduced large-scale communication among cortical regions by decreasing oscillations and coherent activity in the gamma frequency range, shifted networks activity towards slow alpha rhythm, decreased MMN peak response and enhanced aberrant higher gamma neuronal network oscillations. Altogether, our data show that acute and chronic ketamine elicited differential changes in network connectivity, ERPs and event-related oscillations (EROs), supporting possible underlying alterations in NMDAR–GABAergic signaling. The findings underscore the relevance of intermittent dosing of ketamine to accurately maintain the functional integrity of neuronal networks for long-term plastic changes and therapeutic effect.

## Introduction

Increased awareness on limitations to treat depression with conventional monoamine-based therapy and the recent evidence of fast-onset antidepressant response to ketamine in depressed patients has led the shift in research for novel mechanisms of action and development of novel class of antidepressant compounds.^1, 2^ Accordingly, ketamine has recently received a ‘Breakthrough therapy’ designation by the Food and Drug Administration (FDA) for intranasal administration for depression, which means that reviews and approvals will be fast-tracked. Preclinical studies have similarly shown that ketamine has robust antidepressant properties in behavioral animal models of depression.^3, 4, 5^ Ketamine acts as a non-competitive NMDAR antagonist with dose-dependent multipotent properties. At the same time, it is a drug of abuse with hallucinogenic properties, which reduced frontal gray matter volume in subjects after chronic use that may have connotation of neocortical networks impairments.^6^ Abnormalities in cortical networks may result in sleep disturbances including shortened non-rapid eye movement (NREM), sleep onset latency, increased REM density, overall disturbance of sleep continuity and reduced slow-wave activity.^7^ Similar to the existing rapid-acting interventions such as, sleep deprivation and electroconvulsive therapy, ketamine enhanced slow-wave activity during NREM sleep and increased levels of brain-derived neurotrophic factor (BDNF), considered as putative central and peripheral surrogate biomarker of synaptic plasticity.^8, 9, 10^ The established relationship between sleep, depression and antidepressant medications and paucity of preclinical reports on effects of ketamine on sleep–wake cycle in rodents, were a motivation to investigate the direct influence of ketamine on the sleep–wake cycle in rats.

In schizophrenia, an abnormality in glutamatergic neurotransmission is believed to explain negative and cognitive symptoms better than the dopamine hypothesis.^11^ Supporting evidence indicate that NMDAR antagonists such as ketamine and phencyclidine (PCP) elicit symptoms and cognitive deficits in healthy men similar to those found in schizophrenia. Ketamine appears to induce hallucinatory and delusional symptoms along a gradient of intensity.^12, 13^ In rodents, sub-anesthetic doses of ketamine (5–10 mg kg^−1^) are commonly used to feature schizophrenia-like symptoms in rodents such as hyperlocomotor activity and to impair cognitive and perceptive functions.^14^ Another important core feature of schizophrenia has been related to dysfunctions in the gamma aminobutyric acid (GABA) system. Modulation of the NMDAR function has an immediate effect on local interactions between inhibitory interneurons and superficial pyramidal cells. As a dissociative anesthetic, ketamine acts through an inhibitory GABAergic mechanism to directly activate α6β3δ and α6β2δ subtypes of GABA_A_ receptors^15^ and decreases the activity of cortical GABAergic interneurons, whereas pyramidal cell firing increases, leading in a net excitation effect elicited by disinhibition of pyramidal cells,^16^ which drive networks into aberrant gamma oscillations. At the anatomical level, pharmacological blockade of NMDAR or their genetic ablation in parvalbumin (PV)-expressing GABAergic interneurons can induce schizophrenia-like behavior in animals.^17, 18, 19, 20^ At the functional level, preclinical and clinical evidence demonstrate that blockade of NMDAR result in increased gamma oscillations via disinhibition of pyramidal cells,^16, 21^ suggesting that gamma network oscillations represents a useful functional tool for understanding alterations in cortical excitation–inhibition balance.

Although network gamma oscillations are relevant mechanisms in cognitive processes, this rhythm is modulated by lower frequency oscillations such as the theta rhythm, and the interplay between those rhythms has received much more interest in contemporary cognitive neuroscience studies.^22, 23^ Both coherence and phase–amplitude coupling (PAC), where the phase of a slower activity determines the amplitude of higher frequency oscillation dynamics, have been proposed as relevant mechanisms mediating connectivity between distant and regional networks and subserving neuronal computation and information processing.^22, 24^ Coordination and synchronization of regional brain networks are required for the integration of sensory input with stored memory information.^25, 26^ Available evidence strongly implicates the NMDAR in the generation of MMN, which is a pre-attentive component of ERP-reflecting sensory memory traces.^27, 28^ Deficits in MMN generation in schizophrenic patients can be understood as abnormal modulation of NMDAR-dependent plasticity that is correlated with the degree of memory dysfunction^29, 30, 31^ and ketamine significantly reduced the magnetic counterpart of MMN, MMNm, in healthy volunteers.^32^

These properties of NMDAR blockade offer useful opportunities to understand functional effects of ketamine on cortico-cortical and cortico-hippocampal large network, motor activity, cognitive information processing.

## Materials and methods

### Animals, surgical and recording procedures

All experimental procedures were conducted in strict accordance with the guidelines of the Association for Assessment and Accreditation of Laboratory Animal Care International (AAALAC), and with the European Communities Council Directive of 24th November 1986 (86/609/EEC) and were approved by local ethical committee. Male Sprague Dawley rats weighing (250–270 g) at the time of surgery were housed in individually ventilated cages located in a sound-attenuated chamber maintained under controlled environmental conditions.^33^ Inclusion and exclusion criteria are part of the standard operating procedure required to include an animal in an experiment and analysis: good health scores monitored by laboratory animal medicine assistants and verified by internal veterinary, good quality EEG signals, and wash-out period of at least 2 weeks in case of animal re-use, body weight of animals <600 g. Only animals that fulfill all these criteria were used in the experiments and analysis.

### Polysomnography study

The surgery was performed using the protocol described earlier.^33^ Under isoflurane anesthesia, steel screws were epidurally placed stereotactically over the right frontal cortex (AP+2 mm, L 2 mm from Bregma), and parietal cortex (AP −6 mm from Bregma, L 3 mm lateral to the midline). For the recording of the electro-oculogram (EOG) and electromyogram (EMG), stainless steel wires (7N51465T5TLT, 51/46 Teflon, Bilaney, Düsseldorf, Germany) were placed in peri-orbital and inserted into the nuchal muscle, respectively. Electrodes were fitted into an 8-hole connector and were fixed with dental cement to the cranium. After surgery, animals were given analgesics and allowed a 2 weeks recovery and adaptation to the recording conditions, after which the EEG, EOG, EMG signals and body movement activities were monitored for 20-h (saline day in all animals). The following recordings were performed for the same duration and circadian conditions following the administration of vehicle and ketamine (2.5, 5 and 10 mg kg^−1^; *n*=32: 8 animals per condition). All recorded signals were digitized at sample rate of 200 Hz and were high-pass filtered with 0.5 Hz filter, and the notch filter at 50 Hz was used to discard power line interference.

Offline, the discriminative analysis used classification rules to assign six vigilance states as being indicative of active wakefulness, passive wakefulness, light sleep, deep sleep, intermediate stage or REM sleep. Additional sleep–wake parameters, such as amount of time spent in each vigilance state, latencies to sleep states onset, sleep periods and the number of transitions between vigilance states were determined.

### EEG network oscillations and connectivity study

The surgery was carried out according to the earlier protocol.^34^ Animals were equipped with six stainless steel fixing screws for the recording of EEG from multiple locations along the antero-posterior axis (frontal left ‘FL’, parietal left ‘PL’, occipital left ‘OL’ and frontal right ‘FR’, parietal right ‘PR’, occipital right ‘OR’, respectively). All electrodes were placed stereotaxically (FL/FR: AP +2 mm, L ±2 mm; PL/PR: AP −2 mm, L ±2 mm and OL/OR: AP −6 mm, L ±2 mm from the Bregma) and referenced to the same ground electrode place midline above of the cerebellum). In addition, stainless steel wires were placed in the muscle of the neck to record the electromyogram activity (EMG). All electrodes were fit into a 10-hole connector then the whole assembly was fixed with dental cement to the cranium. General motor activity was measured in the home cage by two passive infrared (PIR) detectors placed above each recording cage, and the envelope activity was used to analyze motion levels. Only continuous waking artifact-free 4-s epochs with low-voltage fast EEG activity, high to moderate EMG and body activities were used in the analysis. After a stable recording baseline of 30-min, signals from the six brain areas were recorded for 2-h at 2 kHz sample rate, digitized with 24 bit resolution and band-pass filtered between 1 and 100 Hz.

### EEG frequency oscillations

Analysis was performed using a method described earlier.^34^ In brief, spectral density estimates were constructed from overlapping windows of 4-s data and plotted for post-drug periods windowing over 1–100 Hz. Power estimates for each frequency band were averaged for each 15-min period for: delta band (1–4 Hz), theta band (theta1: 4–6.5 Hz, theta2: 6.5–8 Hz), alpha band (alpha1: 8–11 Hz; alpha2: 11–14 Hz), beta band (beta1: 14–18 Hz; beta2: 18–32 Hz), gamma band (gamma1: 32–48; gamma2: 52–100 Hz). Drug-induced changes in EEG spectral power was calculated in blocks of 15-min for 2-h as the ratio of mean spectral power obtained following once daily administration during 4 weeks of ketamine before the start of the recording session versus the mean spectral power obtained during 30-min baseline period.

### Phase–amplitude cross-frequency coupling

The nonlinear PAC via modulation index (MI) is a powerful index to estimate changes in the strength of interactions between the phase of low-frequency oscillations and the amplitude of higher frequency oscillations. Here, we have used the algorithm described previously,^35^ to analyze PAC values for each EEG electrode in consecutive intervals of 5 min length during baseline and post-drug periods. MI was estimated between low frequencies (‘phase’) *f*_L_ taken from interval 2–12 Hz with a step of 2 Hz and frequencies (‘amplitude’) *f*_H_ taken from interval 10–200 Hz with a step of 5 Hz. MI is reported as absolute value of mean (over time) of complex-valued signal *z*(*t*)*=A*_H_(*t*)·exp(*i*·*ϕ*_L_(*t*)), where *t* is time, *A*_H_(*t*) is analytic amplitude obtained via Hilbert transform of narrow band-pass signal *x*_H_(*t*) centered around frequency *f*_H_, and *ϕ*_L_(*t*) is analytic phase obtained via Hilbert transform of narrow band-pass signal *x*_L_(*t*) centered around frequency *f*_L_. Normalization and statistical assessment (*α*=0.05) were done through construction of 50 surrogates and using Bonferroni correction.^35^ Only statistically significant results were reported on comodulogram maps.

### Networks connectivity

Coherence analysis was used to estimate effects of ketamine on the functional coupling between different cortical structures at various frequencies.^24^ Coherence between the time series in different electrodes was quantified at different time points before and after administration of drugs using a generalized additive mixed model, where the coherence over the frequencies was fitted using a cubic spline for the different treatment groups and the heterogeneity between the animals.^34^ Subsequently, coherence between each pair of electrodes was concatenated every 4-s and aggregated over 15-min in each frequency bands for each animal, time point and treatment group. Particular frequency bands were selected for comparison between groups based on the most prominent oscillatory and coherent activities across different brain regions. To reduce the impact of volume conduction of signals propagated from common generators, all edges in which the maximum absolute value of coherence occurred at zero time lags between time series were removed.

### Auditory passive oddball paradigm: mismatch negativity ‘MMN’ study

All surgery was performed as previously described.^36^ Briefly, animals were instrumented with six active electrodes over the right and left hemispheres (auditory cortex: 2 mm posterior to Bregma and ±2 mm lateral to the midline; parietal cortex: −3 mm posterior to Bregma and ±4 mm lateral to the midline; the occipital cortex −5.5 mm posterior to Bregma and ±4 mm lateral to the midline) and referenced to the ground electrode placed on cerebellum. Electrodes were connected as above into a 10-hole connector and fixed with dental cement to the skull. After a recovery period of 2 weeks, a baseline EEG was recorded for 20-min without acoustic stimuli, ERPs to auditory stimuli were recorded for 20-min without drug treatment followed with additional two runs of 20-min after ketamine administration (2.5, 5 and 10 mg kg^−1^). Auditory stimuli were generated with a custom program written in LabVIEW and sound stimuli were delivered through a speaker mounted above the floor of the experimental box. Sound intensity was calibrated with a sound meter 75 dB SPL in different locations of the recording box. In this passive oddball paradigm, two stimulus conditions (standard and deviant) were pseudo randomly presented with the restriction that consecutive deviants were separated by at least three standards: a series of 240 frequent standard tones 2 KHz tone (probability 80%) and infrequent 60 oddball deviant 4 KHz tones (probability 20%), with intensity 87 dB SPL and 20-ms duration with interstimulus interval of 4-s. Intervals of EEG recordings spanning time from 50-ms before to 450-ms after stimulus onset were extracted, baseline-corrected and averaged ERP waveforms were computed for each animal and stimulus condition, whereas average across animals resulted in grand average ERPs. The peak amplitudes and latencies of the P1, N1, P2 and N2 components of the ERPs were calculated for each rat and condition. In addition, spectrograms averaged across animals were estimated using Morlet wavelet analysis for each stimulus condition and for periods before and after drug treatment.

### Drugs

Ketamine HCl was dissolved in physiological saline solution to achieve a final concentration of 0.25, 0.5 and 1 mg ml^−1^ and has been administered subcutaneously (s.c.) in a volume of 5 ml kg^−1^ body weight. Saline was administered in control animals.

### Statistics

All data were presented as means±s.e.m. A mixed-model design ANOVA was used to analyze the changes in time spent in vigilance states and related variables. Data were checked for normality by applying the Shapiro–Wilk test and homogeneity of variances through the Levene’s test. Time-course changes in network frequency oscillations were estimated by a two-way multivariate ANOVA for repeated measures with two main factors (treatment and period of recordings), followed by a one-way ANOVA with main factor treatment for each of the 15-min period. For network connectivity, the aggregated coherences and changes from baseline were analyzed per frequency band using an ANOVA with time, group and its interaction as covariates while taking into account the heterogeneity between the different animals.^34^ *P*-values from the associated F-tests were reported as well as least squares means from the corresponding ANOVA models. The MMN response, differences in the latencies and amplitudes of the N1 component were determined using a one-way analysis of variance (ANOVA, Turkey post-test).

## Results

### Effects of ketamine on sleep–wake cycle

**Figure 1:**
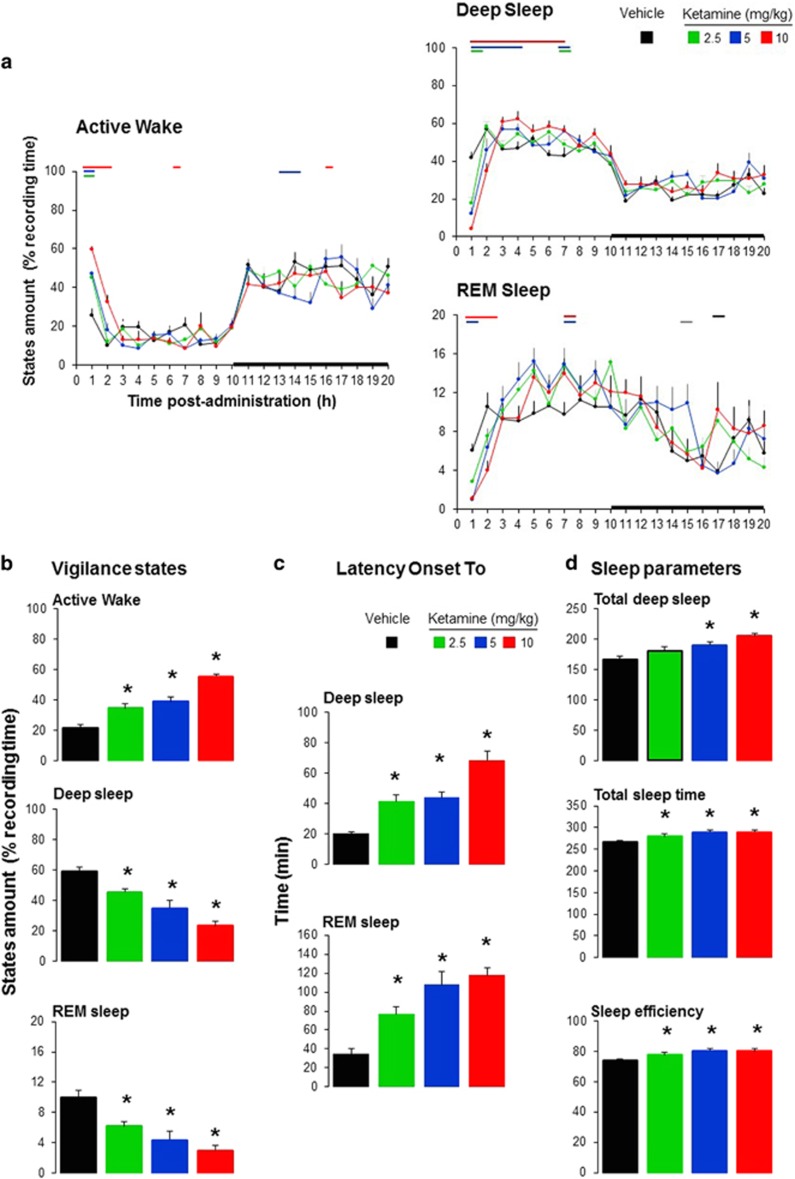
Effects of subcutaneous administration of ketamine (2.5, 5, 10 mg kg^−1^) or saline on (**a**) waking, deep sleep and rapid eye movement (REM) sleep during 20 consecutive hours. (**b**) Active waking, deep sleep and REM sleep during the first 2-h post-administration. (**c**) Latency to deep sleep and REM sleep states. (**d**) Total deep sleep, total sleep time and sleep efficiency in the next 6-h after the initial arousal effect. Dark square area represents the dark period. Lines above curves and stars above bar graphs indicate interval difference from vehicle, respectively (mixed-model design ANOVA).

Ketamine at the different doses tested consistently modified the sleep–wake architecture in rats (Figure 1a). The maximal enhancement of active waking occurred during the first 2-h after subcutaneous administration. Ketamine exerted a wakefulness-promoting effect (treatment × time effect, F_(9, 81)_=3.04, *P*=0.003) for up to 2-h following dosing associated with reduced duration of deep sleep (treatment × time effect, F_(9, 81)_=7.91, *P*<0.001) and REM sleep (treatment effect, F_(3, 27)_=12.3, *P*<0.001) and (time effect, F_(3, 81)_=27.3, *P*<0.001), although the interaction did not reach significance level (treatment × time effect, F_(9, 81)_=1.6, *P*=0.13) (Figure 1a).

Subsequent sleep inhibition resulted in rebound sleep in the next 6-h expressed by an increase in deep sleep time (treatment effect, F_(3, 27)_=7.3, *P*=0.001), total sleep time (treatment effect, F_(3, 27)_=5.8, *P*=0.003) and sleep efficiency (Figure 1c). Mean duration of deep sleep bouts was significantly enhanced, whereas the number of deep sleep bouts was reduced.

The arousal promoting effect was supported by a significant dose-dependent increase in sleep latencies (Figure 1b), whereas no consistent changes were found in total sleep time and sleep efficiency (Figure 1c).

#### Effects of acute ketamine on networks oscillations and motor levels

**Figure 2:**
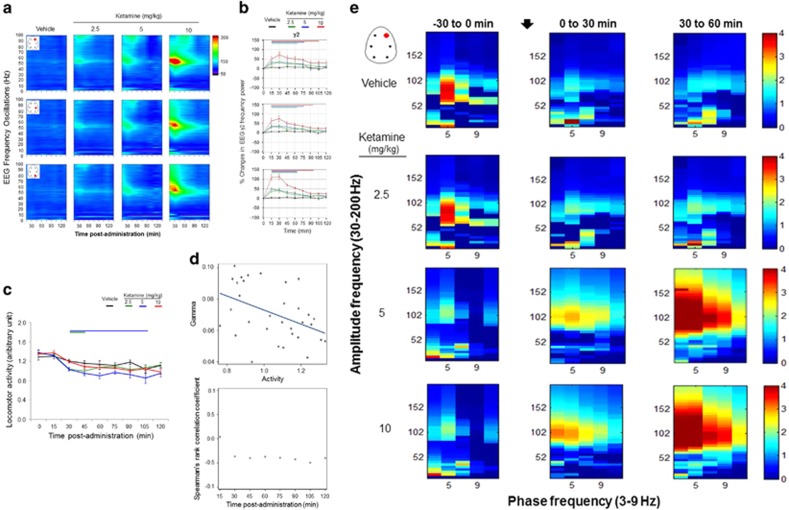
(**a**) Full-power spectrum expressed in heat maps in fronto-parieto-occipital cortical areas during each 15-min block of the 2-h post-administration of ketamine (2.5, 5, 10 mg kg^−1^). Only right hemisphere locations along the antero-posterior axes were presented (frontal right ‘FR’, parietal right ‘PR’, occipital right ‘OR’, see red dots on the brain diagram). Changes in color from cold dark blue to warm red color indicate an order increase in the magnitude of oscillatory power. (**b**) Changes in gamma power during the first 2-h post-administration of ketamine. Color coded bars underneath the curves indicate intervals in which oscillatory activity differed from vehicle (mixed-model design ANOVA). (**c**) Locomotor activity during the first 2-h post-administration of ketamine. (**d**) Spearman correlations (upper panels) at 30-min after the administration of ketamine between the gamma oscillations and locomotor activity and (lower panels) Spearman’s rank correlation coefficients (*R*-values) plotted for each time block of 15-min. (**e**) Time course of phase–amplitude cross-frequency coupling (CFC) for pre- and post-administration of vehicle and of ketamine (2.5, 5, 10 mg kg^−1^). The comodulograms show coupling strength between the phase of theta frequency and the amplitude of high-gamma activity. During baseline period, phase–amplitude coupling (PAC) was observed between theta (5 Hz) and high-gamma (100 Hz). Ketamine at the 5 and 10 mg kg^−1^ elicited a shift in the modulation index (MI) of phase-frequency coupling from theta towards delta and an increase in the strength of MI in broadband from 60–150 Hz with the strongest modulation occurring in the tie interval 30–60 min. MI data are presented as means values in the frontal right structures.

Acute ketamine consistently increased higher gamma (52–100 Hz) oscillations in different brain areas during the first 30-min, after which the power levels gradually returned to basal levels (Figure 2a). Ketamine at the doses of 2.5 and 5 mg kg^−1^ elicited similar aberrant pattern in higher gamma oscillations. However, ketamine at the higher dose of 10 mg kg^−1^ elicited pronounced aberrant high-gamma levels. Quantitative assessment showed a pronounced effect on higher gamma frequency waves in parietal and occipital cortical areas, whereas a negligible effect was observed on theta frequency waves (Figure 2b).

Correlations between gamma oscillations at different time points with related activity levels are shown in Figure 2c. The activity levels showed a steady decrease after the administration of different doses of ketamine administration. The results of a Spearman-rank correlations analysis at 30-min after the administration of ketamine showed negative correlations between higher gamma oscillations and activity levels (ketamine: *r*=−0.3, *P*=0.02). A significant negative association between EEG high-gamma oscillations and activity for each time intervals is indicated in the scatter plot in Figure 2d right bottom panel (Spearman-rank correlation analysis for all time points: rS ranges between −0.3 and −0.5, all *P*-values<0.05).

#### Effects of acute ketamine on cross-frequency coupling

During basal conditions (−30 to 0 min before drug administration), PAC analysis of EEG recordings at the frontal sites showed that phases in slow theta range modulated the amplitudes of high-gamma frequency band (Figure 2e). The administration of ketamine induced changes in both gamma and low-frequency oscillations including theta activity. To understand the relationship between those oscillatory patterns, we have examined the degree of interactions by computing the phase-to-amplitude coupling in those rhythms. In the first time interval (0 to 30 min post-drug administration), when ketamine enhanced the power in higher gamma oscillation, phases of slow theta oscillations more strongly modulated the amplitude of gamma frequency around 100 Hz, where strength depends on the dose. In the subsequent time interval (30 to 60 min), a marked increase in the strength of coupling is particularly observed at the frontal recording sites for high doses (Figure 2e) at the time when gamma power was progressively decreasing (compare with Figure 2b). This strength of interactions was shifted significantly to the delta frequency oscillations that entrained wider higher gamma frequency range (Figure 2e).

#### Effects of chronic ketamine on networks oscillations and connectivity

**Figure 3:**
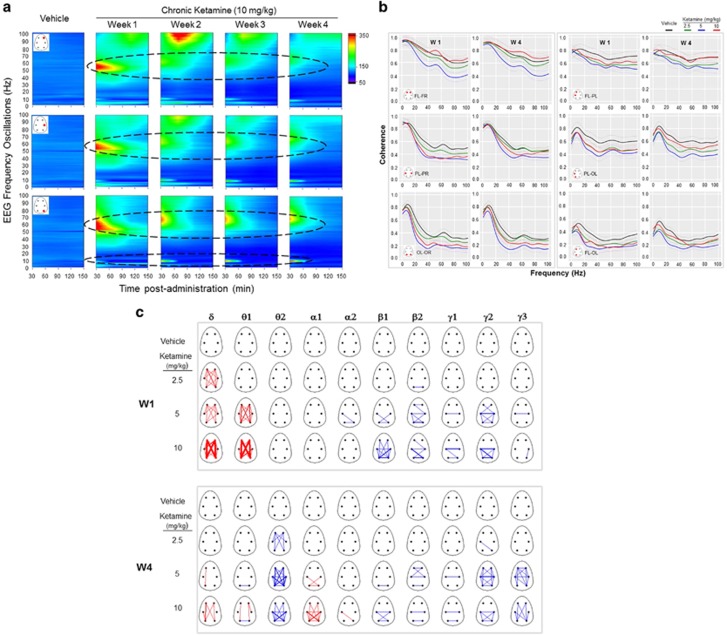
(**a**) Full-power spectrum expressed in heat maps in fronto-parieto-occipital cortical areas during each 15-min time block after once daily chronic administration of ketamine (10 mg kg^−1^). Only right hemisphere locations along the antero-posterior axes of the first day of each week (W) are displayed for (frontal right ‘FR’, parietal right ‘PR’, and occipital right ‘OR’). Changes in color from cold dark blue to warm red color indicate an order increase in the magnitude of oscillatory power. Note the increased higher gamma power following acute administration of ketamine was followed by a consistent shift in power towards slow alpha rhythm during repeated treatment. (**b**) Coherence indices over 0.5–100 Hz at 30-min after repeated administration of ketamine (2.5, 5 and 10 mg kg^−1^) and vehicle. W1 and W4 indicate weeks 1 and 4 following repeated treatments. Ketamine decreased coherence in different oscillatory activities across all selected cortical pairs in comparison with that of the control group. (**c**) Functional network coherence changes from baseline in each frequency band of interest derived from consecutive 4-s epochs concatenated and averaged in 15-min. The width of the edges between pair electrodes is drawn proportional to the weight of changes in coherent activity during baseline session from baseline (0–50%, 50–100% and 100–150%). Red color represents increases and blue color indicates decreases. Average networks derived from the first recording session after ketamine treatment for each frequency band. Time-evolving dominant coherent activity in the frontal–parietal and occipital structures in δ/θ1 and α1 indicated the most persistent edges scaled to the frequency of the most common edge during waking in W1 and W4, respectively.

**Figure 4:**
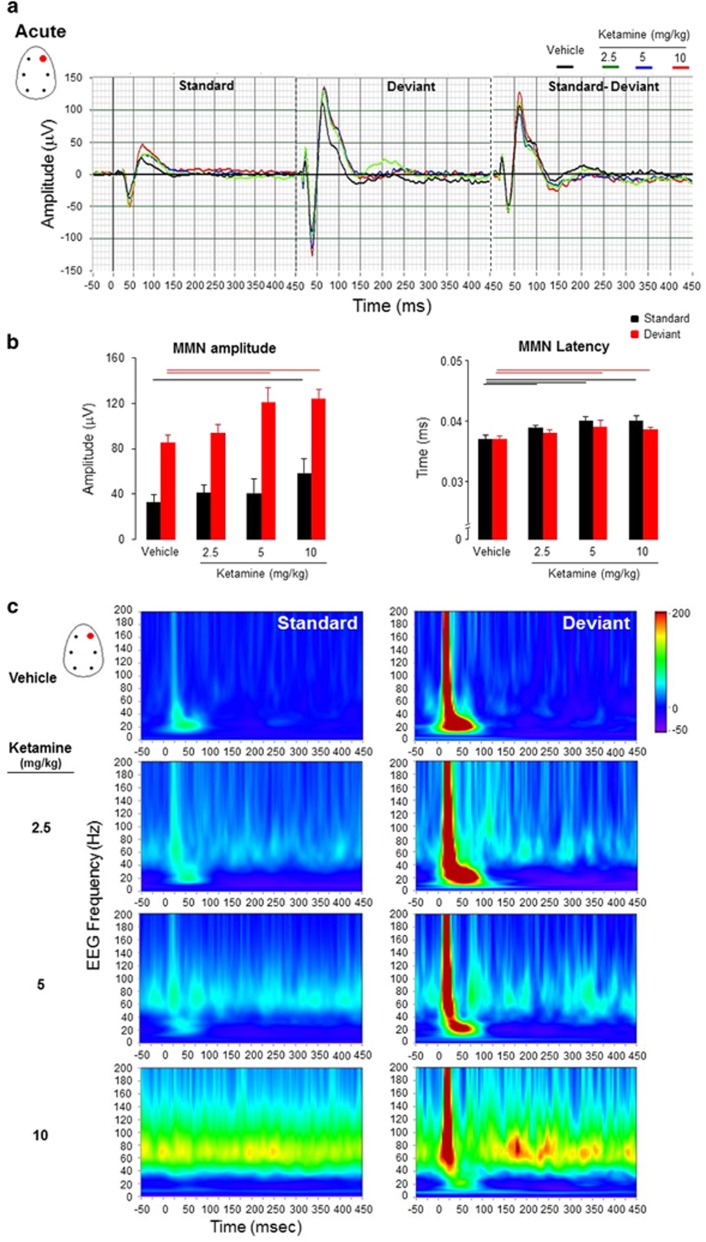
(**a**) Grand-averaged auditory-evoked waveforms from the frontal right electrodes in the frequency deviants’ passive oddball paradigm with obtained after acute administration of ketamine (2.5, 5 and 10 mg kg^−1^) and of vehicle. (**b**) Peak mean amplitude of N1 component (uV) and its latency (ms) are presented for standard and deviant stimuli (*n*=7–8 for each pharmacological condition). Note that acute ketamine, particularly at the higher dose, enhanced amplitudes to both standard and deviant acoustic stimuli suggesting encoding and discrimination facilitation of the sound features during information processing. The horizontal lines above the bar graphs mark significance level (black color coded line for standards and red for deviants; linear mixed-model design ANOVA). (**c**) Phase-evoked and -induced power time-frequency maps at the frontal right electrode in response to standard and deviant stimuli.

Chronic ketamine decreased the high-gamma oscillations and shifted the networks activity towards slow alpha rhythm (Figure 3a, only the dose of 10 mg kg^−1^ of ketamine was displayed). This effect was observed on week 1 after administration and persistently remains across 4 weeks following chronic administration. The simultaneous evoked abnormal alpha and gamma oscillations likely underlie the mechanism mediating memory dysfunctions following sustained NMDAR blockade (Figure 4).

Assessment of coherent activity across different cortical areas at 30-min intervals post-administration of different doses of ketamine revealed a consistent reduction coherent activity across the full-frequency spectrum in different cortical pairs (Figure 3b). The decrease in coherence across the long axis was higher with increasing distance between electrode pairs. A slight increase in coherence between 40 and 60 Hz was found with the higher dose in fronto-frontal electrode pairs.

Time-evolving functional connectivity indicates that administration of ketamine on week 1 significantly increased coherent slow delta and theta frequency activity ipsilateral and contra lateral in frontal, parietal and occipital cortical regions, whereas a consistent decrease in coherent activity was found from beta to higher gamma frequency bands (Figure 3c, W1). After 4 weeks of ketamine administration, an enhanced coherent slow alpha activity was found ipsilateral and contra lateral in frontal, parietal and occipital cortical regions (Figure 3c, W4).

#### Effect of acute and chronic ketamine on mismatch responses to frequency-deviant stimuli

The MMN is an electrophysiological indicator of the pre-attentive stages of short-term memory involved in sensory–auditory information processing. In the auditory cortex, a detectable change (deviant stimulus) in the pitch frequency of a repeated (standard) stimulus evoked a prominent negative polarity in the auditory potential component within a 30–50 ms time window from stimulus onset, likely reflecting a MMN-like response under vehicle treatment (Figure 4a).

##### Acute ketamine

There was no treatment effect to standard stimuli F_(3, 26)_=1.30, *P*=0.3). *Post hoc*analysis showed that ketamine at the dose of 10 mg kg^−1^ enhanced the peak amplitude of the N1 component in response to standard stimuli *P*=0.049 (Figures 4a and b). In addition, there was a treatment effect on peak-amplitude latencies to the N1 component (F_(3, 26)_=5.17, *P*=0.006) (Figure 4b).

There was a treatment effect on the peak amplitude of the N1 component for deviant stimuli F_(3, 26)_=4.30, *P*=0.014). N1 peak-amplitude latencies were enhanced at the doses of 5 and 10 mg kg^−1^ (*P*=0.02 and *P*=0.046, respectively).

The increased response to acoustic standard stimuli, particularly with the high dose of ketamine, suggests facilitation of encoding or new learning material, whereas enhanced MMN response may indicate facilitation of recall and discrimination of deviant acoustic stimuli, whereas the increased peak-amplitude latencies to the N1 component to both standard and deviant stimuli may suggest a slow speed of cortical processing.

The evoked and induced oscillatory pattern was different in vehicle- and ketamine-treated animals (Figure 4c). A sustained gamma band activity mainly between 40 and 100 Hz was the most relevant component associated with MMN response for up to 450 ms post-stimuli, particularly with the higher dose of ketamine.

##### Chronic ketamine

There was no treatment effect to standard stimuli F_(3, 26)_=2.63, *P*=0.07). *Post hoc* analysis showed that ketamine at the dose of 2.5  and 10 mg kg^−1^ reduced significantly the peak amplitude of N1 component (*P*=0.03 and *P*=0.02, respectively), whereas effect at the dose of 5 mg kg^−1^ did not reach significance level. No treatment effect was found for latencies to standard stimuli F_(3, 26)_=2.27, *P*=0.1), whereas *post hoc*analysis showed that ketamine at the high dose of 10 mg kg^−1^ increased the latency to peak amplitude of the N1 component (*P*=0.01).

**Figure 5:**
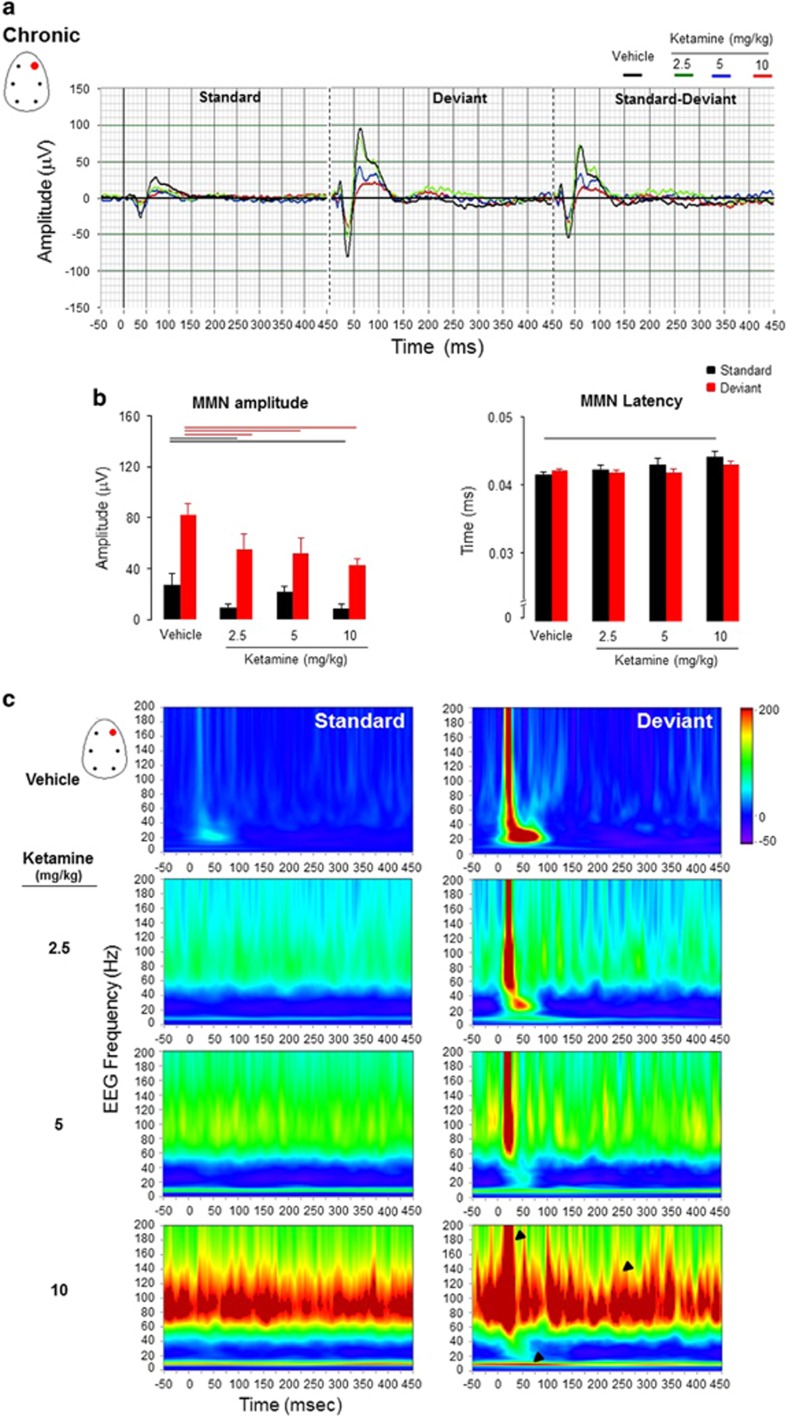
(**a**) Grand-averaged auditory-evoked waveforms recorded from the frontal right electrodes in the frequency deviants’ passive oddball paradigm obtained after chronic treatment with ketamine (2.5, 5 and 10 mg kg^−1^). (**b**) Peak mean amplitude of N1 component (uV) and its latency (ms) are shown for standards and deviants stimuli (*n*=7–8 for each pharmacological condition). The horizontal lines above the bar graphs mark significance level (black color coded line for standards and red for deviants; linear mixed-model design ANOVA). Note that ketamine, particularly at the higher dose, decreased N1 peak amplitude and increased its peak latency to both standard and deviant stimuli. (**c**) Phase-evoked and -induced power time-frequency maps at the frontal right left electrode in response to standard and deviant stimuli. Chronic ketamine enhanced phase-locked slow alpha and high gamma frequency oscillations with the exogenous stimuli (first arrow at the dose of 10 mg kg^−1^) followed by induced aberrant gamma oscillations (second arrow).

A treatment effect was observed for deviant stimuli F_(3, 26)_=3.20, *P*=0.03) (Figures 5a and b).

The reduced response to both acoustic standard and deviant stimuli and the increased peak-amplitude latency, particularly with the high dose of ketamine, indicate disruption in encoding features of standard stimuli and recall of memory traces following slow-speed cortical processing.

The evoked and induced oscillatory activities in the 40–60 Hz was reduced and lower range frequency alpha neural oscillatory activities was also associated with MMN response, which of abnormalities were sustained for up to 450-ms post-stimuli (Figure 5c).

## Discussion

The present results further support the causal relationship between blockade of NMDAR neurotransmission and changes in vigilance states and networks dynamics. It is hypothesized that intermittent administration of ketamine could overcome the progressive decline in cognitive processing likely ascribed to degenerative fast-spiking GABAergic PV+ cells in hippocampal structures.

### Ketamine elicited biphasic effects on vigilance states

#### Ketamine elicited significant wake-enhancing properties supported by lengthened latency onset to sleep

The brain regions and neurotransmitter systems mediating ketamine-induced waking remain incompletely understood. Blockade of NMDAR is known to promote the release of a variety of neurotransmitters, most of which have important roles in arousal. Ketamine evoked serotonin and dopamine release in the prefrontal cortex,^37, 38^ and histamine release in three limbic brain regions.^39^ Alternatively, the enhanced excitability could result from a prolonged change in glutamatergic signaling-induced firing of GABAergic interneurons, which in turn disinhibits cortical glutamatergic pyramidal cells.^16, 38^ The resulting increase in glutamate release and activation of AMPA receptors culminate in an activity-dependent release of neurotrophic factor BDNF that may potentiate synaptic strength and plasticity.

#### Ketamine elicited longer deep sleep bouts, decreased REM sleep and lengthened its onset latency

Decreased slow-wave sleep and increased REM density are a prominent clinical biomarker of depression.^40, 41^ Most patients classified as ketamine responders had low levels of slow-wave sleep and BDNF at baseline. Increased total sleep time associated with increased levels of BDNF and decreased waking occur during the first and second night post infusion of ketamine, suggesting that these measures are associated with the enduring treatment response.^42^ Earlier study in rats demonstrated that ketamine stimulates slow-wave activity during NREM sleep.^43^ Our results further extend these observations, notably that ketamine lengthened mean duration of deep sleep bouts suggesting maintained and consolidated deep sleep.

Most clinically effective antidepressant drugs produce a robust and immediate suppression of REM sleep in animals, healthy volunteers and depressed patients.^44, 45^ The effects are dose-related reduction in the overall amount of REM sleep and delayed REM sleep onset latency. However, the delayed onset of antidepressant action is a major issue with existing monoamine-based antidepressant therapies and the glutamatergic mechanism-based approach may overcome these limitations. Recent evidence indicate that ketamine at sub-anesthetic doses elicited a robust fast onset (within 2-h) and sustained (for 1 to 2 weeks) antidepressant effects in patients with treatment-resistant major depression and bipolar depression disorders.^46^ Similarly, preclinical reports have shown a robust antidepressant property of ketamine in different behavioral paradigms.^3, 4, 5, 47^ Consistent with these observations, our results show that ketamine reduced REM sleep and prolonged REM latency onset, which may mimic the activity of antidepressant drugs on sleep–wake behavior.

The mechanisms underlying ketamine-induced REM sleep suppression are not clear, but it may involve cholinergic pathways. Cholinergic neurotransmission have an important active role in the generation of REM sleep.^48^ Ketamine and MK801 decreased acetylcholine release in the pontine reticular formation structures,^49^ which are relevant brainstem generators of REM sleep sending cholinergic afferents to basal forebrain and ventromedial prefrontal cortical structures implicated in the pathophysiology of depression. Recently, it has been demonstrated that three different classes of antidepressants such as the tricyclic antidepressant desipramine, selective serotonin re-uptake inhibitor fluoxetine and ketamine selectively activate a common set of structures in the ventromedial prefrontal cortex in rats.^50^ Thus, it is tempting to suggest that reduced acetylcholine tone in cortical areas is associated with antidepressant potential of ketamine. However, ketamine has also been shown to stimulate acetylcholine release in prefrontal cortex, hippocampus and striatum,^51, 52^ suggesting that other players than monoaminergic systems are involved such as the mammalian target of rapamycin (mTOR) complex 1. The mTOR complex 1 has been implicated in activity-dependent synaptic plasticity and is localized in neuronal dendrites and spines where it controls synaptogenesis. The same path has been activated by the muscarinic antagonist scopolamine, which is believed to elicit rapid antidepressant effects in depressed patients.^53^

Here, ketamine elicited an initial arousal effect associated with reduced REM sleep occurrence, which may contribute to its antidepressant action. The subsequent sleep facilitation observed here extends earlier observations suggesting possible strengthening of plastic processes.

### Ketamine alters networks dynamics

#### Acute ketamine enhanced aberrant gamma oscillations independently from changes in motor activity levels

Abnormal network gamma oscillations have been associated with positive, negative and cognitive symptoms of schizophrenia.^54^ NMDAR antagonists, which have widely been used to model symptoms of the disease, enhance EEG gamma rhythm in healthy humans and animals.^21, 55, 56, 57, 58^ Gamma power in the rodent prefrontal cortex increases in fast-moving rats.^59, 60, 61^ Consistent with earlier reports, our results showed that ketamine induced higher gamma oscillations, however the changes occurred at low motion levels, supported by negative Spearman correlations between both variables.

Mixed results were reported on ketamine induced changes in theta oscillations in rodents. Although ketamine enhanced theta frequency band,^62^ other studies found no effect on this oscillatory rhythm.^56, 57, 63^ In line with the later reports, no consistent effect was observed on theta oscillations.

#### Ketamine affects phase–amplitude CFC

Coherent activity and PAC are believed to have a key role in coordinating local and widespread brain neuronal networks to mediate neural and cognitive processing in humans and animals.^22, 35, 64^ Interaction of theta and gamma frequency oscillations is key for coordinating distributed brain areas during information processing. Those oscillatory rhythms are essentially enhanced when animals are engaged in memory and learning cognitive tasks, during exploratory movements, and were potentiated by cognitive-enhancing drugs.^34^ Available evidence strongly indicates a central role of the NMDAR signaling in the alteration of neuronal network dynamics. Here, a marked increase was found in the coupling MI and a shift in phase from theta to delta-modulating broadband gamma frequency from 60 to 150 Hz at the interval time of 30–60 min post-administration. Our results agree and extend recent findings showing an exaggerated increase in the theta-mediated high-gamma frequency coupling elicited by ketamine.^65^

#### Chronic ketamine-attenuated network gamma oscillations

Chronic administration of ketamine decreased coherent gamma frequency activity and shifted the networks activity towards slow alpha rhythm.

A pathological increases in gamma oscillatory activity was common to psychotic symptoms of schizophrenia,^54^ whereas a reduction in gamma oscillations was associated with the severity of negative symptoms, likely resulted from deficiency in temporal coordination of cortical networks.^66, 67, 68^ In healthy men, ketamine has been found to decrease functional connectivity between brain areas such as the posterior cingulate and dorsomedial prefrontal cortex, pregenual anterior cingulate cortex and mediofrontal cortex.^69^

Here, chronic ketamine markedly decreased coherence between different electrodes pairs, which may lead in decoupling of local gamma oscillations from the controlling influence of distributed networks. Consequently, a shift towards cortical hyper excitability of the thalamocortical loop resulted in a pathological increase in coherent alpha oscillatory activity. The data indicate that the long-range hippocampal–frontal interactions were sensitive to ketamine and therefore highly dependent on NMDAR signaling.

#### Acute ketamine enhanced, whereas chronic ketamine reduced MMN generation

MMN provides a real-time course of the brain’s synaptic function underlying pre-attentive novelty detection process in the auditory domain^27^ and index short-term memory traces of the standard stimulus at the moment of presentation of a deviant stimulus. Deficits in MMN amplitude, latency and event-related oscillations represent robust neurophysiological markers in neurological and psychiatric.^28, 31^ MMN activity has been demonstrated in rodents.^30, 70, 71^ Blockade of NMDAR using PCP or MK801 selectively abolished the generation of MMN response.^11, 71^ Thus, the strength of MMN reflects the functional condition of NMDAR signaling, which makes this property of NMDAR suited for studying effects of ketamine on cognitive information processing. Here, acute ketamine facilitated the generation of the MMN response as it was observed with the NMDAR antagonist memantine, which enhanced auditory change detection in human and animals.^71, 72^ Thus, ketamine administered acutely may strengthen encoding and retrieval representation and therefore have transient facilitative effects on the pre-attentive stages of short-term memory processing. However, chronic ketamine blocked the generation of the auditory MMN-like response and therefore may weaken the strength of encoding, retention and retrieval of memories, as it was found following NMDAR blockade.^73, 74^

Abnormal oscillations in the 40–70 Hz frequency range is considered as a putative cause for cognitive deficits in schizophrenia and their first degree relatives because robust reduction were found in auditory steady state potentials and oscillations around 40 Hz, which serves perception and cognition providing a mechanism for temporal binding of neural activities underlying mental representations.^75, 76^ Here, EROs responses underlying the passive oddball paradigm were found in the 40 Hz in acute ketamine-treated animals; however, this oscillatory rhythm was completely impaired and additional oscillatory contribution shifted to lower alpha in chronically ketamine-treated animals. EROs in higher gamma frequencies were maintained during both treatments.

Thus, ketamine-induced changes in evoked networks oscillations, connectivity and cross-frequency coupling may contribute deficits in MMN generation.

Converging lines of evidence implicate dysfunction in synchronization of gamma network oscillations in the pathophysiology of schizophrenia. The parvalbumin interneurons have a key role in the genesis of gamma oscillations in cortical networks as they exert a strong temporal inhibition onto their target pyramidal cells and interneurons.^77, 78^ Reduction or disturbance of NMDAR transmission on inhibitory GABAergic interneurons, which contain the PV-positive cells may contribute to disturbances in gamma network dynamics, and induce schizophrenia-like behavior in animals.^54, 79, 80^ GABAergic fast spiking interneurons maintain a balance of excitation and inhibition in the cortical network and those PV-positive interneurons have a critical role in the induction and maintenance of synchronous gamma network oscillations, required for working memory and cognitive information processing. Post-mortem studies in the brains of schizophrenic patients.^80, 81^ And preclinical pharmacological studies with NMDAR blockers as well as neurodevelopmental animal models of schizophrenia have shown a consistent reduction in the number of PV-positive cells and spine density in the frontal cortex, nucleus accumbens and hippocampus.^82, 83, 84^ The present functional abnormalities may point towards an NMDAR-hypofunction-induced abnormal-balanced functional networks and PV-positive cells.

### Significance

Alteration of cortical excitation–inhibition balance is believed to significantly contribute to the pathophysiology of psychiatric disorders such as depression and schizophrenia. Ketamine has demonstrated robust fast-onset antidepressant efficacy in numerous clinical trials; however, the mechanisms underlying this rapid onset effect are not fully understood. Neurophysiological variables may provide great opportunities for translational research in revealing functional biomarkers. Scopolamine the non-specific muscarinic receptor antagonist and sleep deprivation have demonstrated rapid antidepressant effects.^85, 86^ Sleep deprivation has been shown to decrease functional connectivity in the default mode network in resting brains^69, 87^ and between the right thalamus with the right parahippocampal gyrus, right middle temporal gyrus and right superior frontal gyrus in the resting brain.^88^ Scopolamine has also been shown to consistently reduce interhemispheric coherence in the delta and beta frequency oscillations during the resting state.^89^ In addition, scopolamine, sleep deprivation and ketamine share activation of similar downstream plastic signaling. Disinhibition of pyramidal cells elicits an increase in AMPA dependent signaling, which triggers an increase in phosphorylation of mTOR and synthesis of neurotrophic BDNF factor and synaptogenis^90, 91, 92^ Thus, ketamine induced increases in gamma oscillations and decreased coherent network might represent a potential early neurophysiological indicator of the signaling cascade underlying the rapid antidepressant action.

Consistent with human data, we found that ketamine can elicit increases and decreases gamma network oscillations after acute and chronic treatment, respectively. The loss of the gamma coordination could derive from a shift in theta-gamma PAC towards delta-broadband; or from the reduction of PV-positive interneurons. Here, the key functional observations were changes in EEG oscillations, reduced network connectivity and cross-frequency coupling, which might bridge the gap between abnormal signaling of GABA PV interneurons and the cognitive deficit observed in the MMN response. Interestingly, a recent pharmacological report revealed that the loss of PV interneurons in the prefrontal cortex contributes to the antidepressant-like actions and is also involved in the propsychotic-like behaviors following acute and repeated ketamine administration, which may partially be mediated by the disinhibition of glutamate signaling.^93^ Therefore, the ability to modulate gamma network oscillations and connectivity could be a useful physiological biomarker for abnormalities in the NMDAR–GABAergic PV signaling and for pharmacological intervention in both animals and human.

Collectively, acute and chronic ketamine treatment elicited differential effects on the dynamics of cortical networks supporting possible causal alterations in NMDAR–GABAergic signaling. The findings underscore the relevance of intermittent dosing of ketamine to accurately maintain the integrity properties of neuronal networks for long-term plastic changes and therapeutic effect.
